# Diagnosis and Management of *Amanita Phalloides* Toxicity in the Emergency Department Observation Unit: A Case Report

**DOI:** 10.5811/cpcem.1268

**Published:** 2024-01-09

**Authors:** Matthew Tobias, Mary McGoldrick, Mary Rometti, Jessica Laub, Grant Wei, Denise Fernandez

**Affiliations:** * Rutgers Robert Wood Johnson Medical School, Department of Emergency Medicine, New Brunswick, New Jersey; † Columbia University Irving Medical Center, Department of Emergency Medicine, New York, New York

**Keywords:** *toxicology*, *mushroom*, *amanita*, *case report*

## Abstract

**Introduction:**

Mushroom toxicity is an important etiology of acute liver injury in a patient with gastrointestinal symptoms.

**Case Report:**

We present the case of a male patient presenting to the emergency department (ED) with gastrointestinal distress who was placed under ED observation for elevated liver function tests. During his hospital course, it was revealed he had consumed wild mushrooms believed to be *Amanita phalloides*.

**Conclusion:**

While mushroom ingestion and subsequent toxicity are rare, a high index of suspicion in foraging hobbyists is essential to arriving at the correct diagnosis and directing the patient to the appropriate management.

Population Health Research Capsule What do we know about this clinical entity?

*Mushroom poisoning can result in liver failure and death. Early clinical presentation includes nonspecific gastrointestinal (GI) symptoms that can be easily missed.*

What makes this presentation of disease reportable?

*We identified this case of mushroom poisoning on reassessment of the patient in the setting of an emergency department observation unit (EDOU).*

What is the major learning point?

*Although rare, mushroom toxicity is an important consideration for patients presenting with GI distress and liver injury.*

How might this improve emergency medicine practice?

*Placing persistently symptomatic patients in an EDOU can provide the ideal location to reveal and rapidly act on critical diagnoses.*

## INTRODUCTION

Of the estimated 100 mushroom species that are toxic to humans, *Amanita phalloides* and others containing amatoxin are responsible for 95% of mushroom poisoning fatalities worldwide.^1^ ^,^ ^2^ In fact, one *A phalloides* mushroom often contains α-amanitin exceeding the lethal dose of about 0.1 milligrams per kilogram (mg/kg) of body weight.^3^ ^,^ ^4^ Disturbingly, mushroom species can be easily mistaken for one another, with *A phalloides* (death cap) often misidentified as the harmless paddy straw mushroom (*Volvariella volvacea*).^5^ We report a case of mushroom poisoning with delayed identification, ultimately diagnosed in our emergency department (ED) observation unit after multiple interviews with the patient. We discuss the pathophysiology and current management of mushroom toxicity and highlight the importance of considering this diagnosis in patients presenting with severe acute liver injury.

## CASE REPORT

A 59-year-old male with a history of hypertension and prediabetes presented to the ED in the morning with diffuse, crampy abdominal pain associated with vomiting and profuse diarrhea. His symptoms began five hours prior to arrival. He reported that the previous night he had eaten a dinner of fish and vegetable curry which tasted “off.” Initial vital signs showed a blood pressure of 153/94 millimeters of mercury, pulse of 83 beats per minute, respiratory rate of 18 respirations per minute, a temperature of 98.2° Fahrenheit, and an oxygen saturation of 98% on room air. Physical examination was unremarkable. He lacked any abdominal tenderness, appeared to be euvolemic, and was in no acute distress.

Treatment began with a one-liter bolus of intravenous (IV) normal saline, 8 milligrams (mg) of IV ondansetron, and 15 mg of IV ketorolac. Initial blood analysis is noted in the table. Outpatient blood analysis from the month prior showed normal liver function testing (LFT) and creatinine (Cr) of 0.8. Right upper quadrant ultrasound was ordered and showed hepatic steatosis, as well as cholelithiasis with biliary sludge, but no evidence of cholecystitis. Six-hour repeat basic metabolic panel (BMP) after fluid hydration did not demonstrate any significant change. The patient was tolerating fluids but complained of persistent abdominal pain and watery diarrhea. He was placed in the ED observation unit pending a computed tomography of the abdomen and pelvis (CTAP) with contrast and continued IV hydration.

**Table:** Significant laboratory findings throughout patient’s hospital course.

	Reference range	Units	Initial presentation	6 hours	24 hours	Peak
Potassium	3.7–5.2	mEq/L	5.7	4.5	6.5	
Bicarbonate	23–29	mEq/L	19	18.4	16.7	
Glucose	70–100	mg/dL	216			
Creatinine	0.6–1.3	mg/dL	1.3	1.3	2.2	
Blood urea nitrogen	6–24	mg/dL	27	30		
Alanine transaminase	4–36	U/L	78		1,450	7,000
Aspartate aminotransferase	8–33	U/L	63		996	1734
Alkaline phosphatase	20–130	U/L	110		79	110
Total bilirubin	0.1–1.2	mg/dL	0.6		1.5	3.9
Prothrombin time	11–13.5	seconds				50.7
International normalized ratio	0.8–1.1					4.6

*mEq/L*, milliequivalents per liter; *mg/dL*, milligrams per deciliter; *U/L*, units per liter.

Overnight, the patient’s symptoms continued. The CTAP showed nonspecific fatty stranding of the left adrenal gland, nonspecific fatty liver disease, and partial visualization of cystic lung disease. The morning laboratory analysis at 24 hours began to show significant and worsening abnormalities in BMP and LFT (table). The patient remained hemodynamically stable, with an unchanged exam. Arrangements were made for admission, and general surgery was consulted for acute liver injury.

On repeating his diet history to the consulting team, the patient revealed he used to forage for mushrooms in his home country. In fact, two days before symptom onset, he had eaten several mushrooms from his yard. He felt well afterward; so he consumed additional mushrooms in a fish curry that he later thought was the cause of his symptoms. Upon reevaluation, he reported 8/10 abdominal pain, 15–20 episodes of watery stools, profuse vomiting, anxiety, diaphoresis, and insomnia. Vital signs remained within normal limits, but his repeat abdominal exam showed mild, diffuse tenderness. He had anicteric sclera but lacked jaundice. Medical toxicology service was consulted for management of liver injury secondary to mushroom ingestion, and the patient was able to provide a photograph of the species he had consumed. While the mushroom itself was unavailable for testing, the photograph was reviewed with a mycologist and suspected to be *A phalloides* (Image).

**Image.**
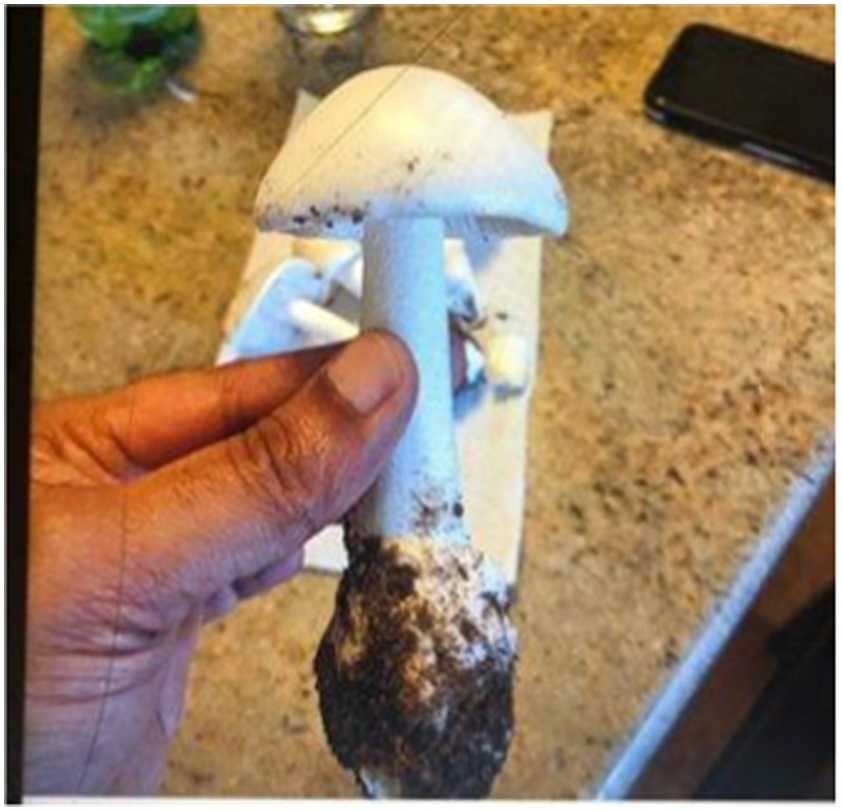
Suspected *Amanita phalloides* mushroom picked by the patient.

Treatment for mushroom toxicity was initiated with N-acetylcysteine (NAC) 21-hour IV protocol (150 mg/kg loading dose over 60 minutes followed by 50 mg/kg over four hours), high-dose IV penicillin at one million units per kg per day (U/kg/day) every four hours, activated charcoal (1g/kg by mouth), and IV hydration. His acetaminophen level, ordered when his worsening transaminitis was identified, was negative. The U.S. Food and Drug Administration’s emergency criteria for silibinin, an amatoxin antidote, were met, and it was initiated on day two as a 5 mg/kg loading dose over one hour followed by a continuous infusion of 20 mg/kg/day. The patient remained without encephalopathic changes. Over the course of several days, his laboratory abnormalities peaked, and their values can be seen in the table. After evaluation by the hepatobiliary service, he was transferred to a regional liver transplant center. Following transfer, treatment with NAC was continued. As LFTs continued to decline, the patient was discharged on hospital day seven without the need for transplant.

## DISCUSSION

Poisoning from amatoxin-containing mushrooms, such as *A phalloides,* is rare worldwide with the highest incidence in western Europe where 50–100 cases are reported annually.^3^ However, this number may be increasing as many people found themselves discovering new hobbies amid the coronavirus disease 2019 pandemic. A recent retrospective review discusses an outbreak of mushroom poisoning in Israel in 2020, while several news reports during the pandemic described an increase in calls to poison control centers after mushroom foraging.^6^ ^–^ ^9^ This increase in foraging among amateur hobbyists may result in higher rates of mushroom poisoning presenting unknowingly to EDs.

Of the 15–20 cyclopeptides contained in *A phalloides,* two toxins are responsible for the majority of symptoms, which explains the different phases of the classic clinical course. The phallotoxin phalloidin is responsible for the initial gastrointestinal dysfunction seen 6-12 hours following ingestion and comprising phase I of toxicity. Based on his initial symptoms, this patient likely presented to the ED during or just after this initial phase of poisoning. Phase III toxicity is hepatic, renal, and neurologic sequelae (such as encephalopathy) caused by the amatoxin α-amanitin, a heat stable ribonucleic acid polymerase inhibitor that is absorbed by hepatocytes and disrupts protein synthesis. This can be seen within 2–6 days following ingestion. In the interim period of phase II, there is short-lived clinical improvement concurrent with the onset of hepatic injury.^3^ Given his increasing transaminitis the morning following presentation, this patient likely was entering the third phase when he was transitioned to the ED observation unit.

Diagnosing mushroom toxicity in the ED relies on thorough history-taking and can be easily missed as earlier symptoms mimic gastroenteritis. Unfortunately, by the time Phase III of mushroom toxicity occurs, a patient may exhibit complications such as hepatorenal syndrome, hepatic encephalopathy, and fulminant liver failure. In the ED, many other potentially fatal diagnoses in the patient with undifferentiated abdominal pain may be revealed with the assistance of abdominal imaging. However, in the case of mushroom toxicity, there is no structural abnormality to be revealed by imaging. Unlike for most cases of gastroenteritis, laboratory testing can be useful for these patients. Increasingly elevated transaminases two to three days following mushroom ingestion is the classic course for amatoxin.^4^ However, while laboratory testing may alert a clinician that something is amiss, a detailed history is vital to uncovering this diagnosis.

Management of the mushroom-poisoned patient is multifactorial. Although there is no single amatoxin antidote, supportive care with fluid and electrolyte repletion is the cornerstone of treatment. Activated charcoal may be of use in blocking toxin reabsorption for a longer time course due to enterohepatic recycling, which continues for up to five days post ingestion.^4^ Animal studies have shown that 1g/kg (1 million U/kg) penicillin G may be beneficial in competing with amatoxin for transport into the hepatocytes. Similarly, NAC is also recommended for its protective effects on the liver, favorable side-effect profile, and impact on mortality. The exact mechanism of action remains unclear for either drug as an amatoxin antidote.^3^ Silibinin, branded as Legalon SIL, is an IV compound isolated from milk thistle extract that competitively inhibits the transporter responsible for the enterohepatic circulation of α-amanitin and may lower the incidence of mortality.^2^ ^,^ ^3^ In cases where these treatments fail, the only remaining therapy is liver transplantation for fulminant hepatic failure.

## CONCLUSION

Mushroom poisoning is an uncommon toxicologic emergency. Symptoms are non-specific and delayed in presentation, while imaging is of little use and laboratory testing may only raise red flags once hepatic damage is already well underway. Emergency clinicians must rely on detailed history-taking to make this crucial diagnosis and institute treatments in a timely manner. Finally, this case highlights the utility of an ED observation unit in identifying diagnoses that may not have been evident due to their nonspecific initial presentation.
